# In Vitro Activity, Stability and Molecular Characterization of Eight Potent Bacteriophages Infecting Carbapenem-Resistant *Klebsiella pneumoniae*

**DOI:** 10.3390/v15010117

**Published:** 2022-12-30

**Authors:** Abeer Ameen Baqer, Kokxin Fang, Norfarhan Mohd-Assaad, Siti Noor Adnalizawati Adnan, Norefrina Shafinaz Md Nor

**Affiliations:** 1 Medical Laboratory Techniques Department, Dijlah University College, Baghdad 10021, Iraq; abeer.ameen@duc.edu.iq; 2 Department of Biological Sciences and Biotechnology, Faculty of Science & Technology, Universiti Kebangsaan Malaysia, Bangi 43600, SGR, Malaysia; kokxinfang@gmail.com; 3 Department of Applied Physics, Faculty of Science & Technology, Universiti Kebangsaan Malaysia, Bangi 43600, SGR, Malaysia; n_farhan@ukm.edu.my; 4 Institute for Systems Biology (INBIOSIS), Universiti Kebangsaan Malaysia, Bangi 43600, SGR, Malaysia; 5 Faculty of Dentistry, Universiti Sains Islam Malaysia, Level 15, Tower B, Persiaran MPAJ, Jalan Pandan Utama, Kuala Lumpur 55100, SGR, Malaysia; adnaliza@usim.edu.my

**Keywords:** bacteriophage, *Klebsiella pneumoniae*, phage therapy, antibiotics alternative

## Abstract

Background: Members of the genus *Klebsiella* are among the leading microbial pathogens associated with nosocomial infection. The increased incidence of antimicrobial resistance in these species has propelled the need for alternate/combination therapeutic regimens to aid clinical treatment, including bacteriophage therapy. Bacteriophages are considered very safe and effective in treating bacterial infections. In this study, we characterize eight lytic bacteriophages that were previously isolated by our team against carbapenem-resistant *Klebsiella pneumoniae*. Methods: The one-step-growth curves, stability and lytic ability of eight bacteriophages were characterized. Restriction fragment length polymorphism (RFLP), random amplification of polymorphic DNA (RAPD) typing analysis and protein profiling were used to characterize the microbes at the molecular level. Phylogenetic trees of four important proteins were constructed for the two selected bacteriophages. Results and conclusions: All eight bacteriophages showed high efficiency for reducing bacterial concentration with high stability under different physical and chemical conditions. We found four major protein bands out of at least ten 15–190 KDa bands that were clearly separated by SDS-PAGE, which were assumed to be the major head and tail proteins. The genomes were found to be dsDNA, with sizes of approximately 36–87 Kb. All bacteriophages reduced the optical density of the planktonic *K. pneumoniae* abruptly, indicating great potential to reduce *K. pneumoniae* infection. In this study, we have found that tail fiber protein can further distinguished closely related bacteriophages. The characterised bacteriophages showed promising potential as candidates against carbapenem-resistant *Klebsiella pneumoniae* via bacteriophage therapy.

## 1. Introduction

The excessive use of antibiotics leads to the emergence of multidrug resistant (MDR) and extensively drug resistant (XDR) bacterial strains. This limits the efficacy of antibiotics in treatments [1] and increases the probability of a patient being recolonized by the resistant strains. The absence of new potent drugs against bacterial infections and the reduced efficiency of currently available drugs are among the largest challenges of the twenty-first century [2]. In 2018, the number of annual deaths worldwide from bacterial infections in the absence of effective antibiotics reached 700 thousand [3]. The projection of the fatality rate is worrying; it is estimated that 10 million people will die from antibiotic-resistant infections in 2050 if the landscape remains similar [4].

The situation is worsened by the fact that the development of new antibacterial agents is nearly absent due to the time-consuming, expensive and complicated process of licensing, in addition to the diminishing pharmaceutical resources and investment withdrawal [5]. The continuous unregulated use of antibiotics has led to the emergence of ESKAPE microorganisms (*Enterococcus faecium*, *Staphylococcus aureus*, *Klebsiella pneumoniae*, *Acinetobacter baumannii*, *Pseudomonas aeruginosa* and *Enterobacter*), which are characterized by their acquired resistance to various antimicrobial agents [1]. Extended spectrum β-lactamase (ESBL) and carbapenemase-producing gram-negative bacteria also pose a challenging therapeutic problem.

Bacteria species from the *Klebsiella* genus can cause numerous community and nosocomial infections, including pneumonia, urinary tract infection, septicemia and wound infection. *Klebsiella pneumoniae* is also one of the major human pathogens that cause respiratory tract infections [6]. Furthermore, *K. pneumoniae* is one of the most frequent agents that contribute to secondary infection events in COVID-19 patients [7].

The resistance prevalence of *Klebsiella* against most commercial antimicrobial drugs has increased exponentially, and pandrug-resistant *Klebsiella pneumoniae* strains/variants have been reported [8]. Currently, the cumulative prevalence of nosocomial multidrug-resistant (MDR) *K. pneumoniae* is estimated at 32.8% [9]. This poses a difficulty for treating these infections with existing antibiotics. The ability of this *Enterobacteriaceae* to gain and transfer antimicrobial-resistance genes, particularly in the health-care settings, represents a critical threat to human health. The economic cost of *Klebsiella* outbreaks in an individual health-care setting is high; for instance, a Dutch hospital reported in 2015 that the cost of an outbreak of an MDR *K. pnumoniae* infecting a total of 29 patients was estimated at $804,263 [10]. Combinational therapies, such as the use of antibiotics together with bacteriophages or other antimicrobial particles, are strongly suggested to treat MDR bacterial infections [11]. Bacteriophages are natural killers of bacteria and, therefore, have the potential to be used as alternative treatments to antibiotics via intravenous administration, as approved by the FDA [12]. Bacteriophage therapy is emerging as a potential weapon against MDR bacterial infections [13]. The discovery of bacteriophage candidates for therapeutic use has been accelerated by bacteriophage characterization and modern sequencing technologies.

In previous bacteriophage studies [14,15], complete information on the stability (storage condition and sensitivity towards environmental factors), in vitro activities (reproduction cycle, optimal infective dose, and adsorption rate) and molecular characterization (protein and DNA analysis) of the bacteriophages was not revealed. Hence, our study exhaustively characterizes the stability, in vitro activities and molecular profile of previously isolated bacteriophages, k2a, k2b, k2w5, k2w6, kp99, k9w5, k9w6 and k9coc, isolated from wastewater, soil and clams against *Klebsiella pneumoniae* [16]. These eight bacteriophages were chosen out of 58 isolates due to their ability to infect and lyse carbapenem-resistant *Klebsiella pneumoniae*. These bacteriophages show consistent morphologies as *Myovirus* and *Podovirus* and hold strong potential for application in bacteriophage therapy, hence the need for their characterization using a combination of traditional and genomic methodologies to understand their infection cycles and susceptibility to physical and chemical agents, as well as their molecular structure. Furthermore, very few publications studied the depolymerase enzyme produced by bacteriophages that causes the formation of halos around their plaques. In our study, we include the effect of time on the diameter of the halos, which also indicate the quantity of enzyme production by different phages.

## 2. Materials and Methods

### 2.1. Bacterial Strains and Culture Conditions

The *Klebsiella* strain used in this study is a carbapenem-resistant *Klebsiella pneumoniae* (KP2) that was described and used in previous work [16]. All culturing in liquid media was performed with shaking (150 rpm) at 37 °C using Nutrient broth or LB broth, as indicated. LB agar and LB-soft agar (0.75% agar) were used for plaque and spot assays.

### 2.2. One-Step Growth Curve

The one-step growth curve was performed according to the method described in [17]. The bacteriophage lysate was added to a prepared bacterial suspension to achieve MOI 0.01. Bacteriophages were allowed to adsorb for 10 min at 37 °C and centrifuged. The obtained pellets were resuspended in SM buffer (1 mL) to remove non-adsorbed bacteriophages, and then diluted in 10 mL of LB medium (1:10,000) and incubated at 37 °C in shaking incubator. At specific time intervals (5, 10, 20, 25, 30, 40, 50, 60, 70 and 80 min), aliquots of 0.1 mL were withdrawn and bacteriophage titers were determined. The method was modified from several studies [15,18]. Experiments were performed on three different replicates and values depict the mean of three observations ± standard deviation. The latency period [14] and burst-size were then determined from the result.

### 2.3. Sensitivity of Bacteriophage to Long-Term Storage, Temperature, Chloroform and pH

For long-term storage, the bacteriophage titer was determined after preserving for 4 months at 4 °C, since it has been reported that the bacteriophage concentration normally decreases with time [19]. For pH-stability testing, 100 μL of the bacteriophage sample (10^8^ PFU/mL) was mixed in a tube containing SM buffer with pH ranging between 2–14 (adjusted using NaOH or HCl) and incubated for 1 h at 37 °C. For thermal-stability testing, 100 μL of the bacteriophage lysate (10^8^ PFU/mL) was mixed with 900 μL SM buffer. Samples were maintained in a water bath ranging between 30–80 °C for 60 min. The method was adapted from [20]. The sensitivity of each bacteriophage to chloroform was tested using 10% chloroform concentration. Chloroform was introduced to 1 × 10^9^ PFU/mL bacteriophage concentration in SM buffer to a final chloroform content of 10% (*v*/*v*). The bacteriophage was shaken to dislodge in the chloroform and allowed to sit for 1 h at room temperature. All samples were withdrawn after 1 h of incubation and checked for bacteriophage titers using KP2 as the host on the double-layer agar [21]. All the experiments were performed in triplicate and the results were taken as the average of the three replicates.

### 2.4. Optimal Multiplicity of Infection (MOI)

To determine the optimal MOI, the bacterial culture was infected with the bacteriophages at 5 different MOI ratios: 0.001, 0.01, 0.1, 1 and 10. The infected cells were incubated for 6 h at 37 °C with mild agitation (170 rpm) after incubation; the suspension was centrifuged for 10 min at 10,000 rpm using Beckman Coulter^®^ Microfuge^®^ 16 Centrifuge to obtain clear supernatants for the bacteriophage titer assay. The MOI with the highest bacteriophage titer was taken as the optimal MOI. All the experiments were performed in triplicate and the results were taken as the average of the three replicates [22].

### 2.5. Bacteriophage Adsorption to Host Bacterial Cells

The rate of adsorption of the bacteriophages to the host bacterial cells was determined by infecting 1 mL of log-phase specific host of *K. pneumoniae* cells (10^8^ CFU/mL) with the bacteriophage suspensions, each at an optimum MOI. The infected cells were incubated at 37 °C for up to 18 min. After 0, 3, 6, 9, 12, 15 and 18 min, two sample batches were withdrawn for each bacteriophage and centrifuged for 3 min at 11,000 rpm to enforce the sedimentation of the bacteria and the adsorbed bacteriophages. The supernatants were sterilized via the passage through a 0.22 μm filter and assayed for un-adsorbed bacteriophages concentration [23]. All the experiments were performed in triplicate, and the average result was taken. The percentages of free bacteriophages and the adsorption rates were calculated following the formula described in [24].

### 2.6. Bacteriophage Structural Protein Analysis by SDS-PAGE

Structural proteins of the eight PEG precipitated bacteriophages were analyzed by SDS-polyacrylamide gel electrophoresis (PAGE). The highly purified bacteriophage isolates (10^10^) PFU/mL were used for bacteriophages protein extraction. A total of 0.5 mL bacteriophage suspension was treated with equal volume of ethanol 96% and incubated at −20 °C for 24 h. After incubation, the ethanol layer was removed by centrifugation the samples at 13,000 rpm for 1 h. The protein precipitates were resuspended in 100 μL of 4% urea and stored in −20 °C. Equal volume of bacteriophages proteins and lysis buffer were mixed and heated in a boiling water bath for 10 min. The samples were then subjected to SDS-PAGE using 12% acrylamide [25]. Protein bands were visualized by staining the gel with Coomassie brilliant blue.

### 2.7. DNA Extraction, Restriction Analysis and RAPD

Prior to the DNA extraction, DNase and RNase were added to the purified bacteriophage lysates and the mixtures were incubated at 37 °C for 1 h. Bacteriophage DNA was extracted and purified from the bacteriophage lysates using phenol extraction and ethanol precipitation [21]. DNA restriction analysis was carried out with 2–5 enzyme units (EcoRI, smaI, xbaI, NdeI, XhoI and PstI) and 1 µg DNA in the reaction mixture. The volume was made up to 20 µL with distilled water and appropriate enzyme buffer according to the recommendations of the manufacturer before incubation, with optimum temperature 30 °C for smaI and 37 °C for the rest of the enzymes for 6 h. An aliquot of the digestion product was resolved by gel electrophoresis. The RAPD method of polymerase chain reaction involves the use of short primers (8–12 nucleotides) of arbitrary sequences for producing unique and reproducible bands that will be utilized for quick bacteriophage typing and preliminary genetic diversity assessment [26]. The five primers used in this study are listed in Table 1. The RAPD-PCR dendrogram was generated using the group linage method using SPSS Hierarchical Cluster analysis.

### 2.8. Bacteriophage Genome Sequencing

Next generation sequencing of the two purified bacteriophages genomic DNA (k9w6 and k7w9) was performed by Apical scientific using the Illumina Hiseq platform at 2 × 150 bp. The library was prepared using NEBNext^®^ Ultra™. The sequence data was assembled and annotated by Malaysian Genome Institute. Gene prediction was carried out using Phanotate [28], which employs a gene calling method tailored for bacteriophage genomes. Predicted genes were annotated using Blast2GO [29], a universal tool for annotation, visualization and analysis in functional genomics to produce BLAST hits, InterPro domain matches, Gene Ontology information, enzyme codes and many more. The top BLAST hit was used to annotate the function of each gene/protein, and those with no hits were annotated as novel proteins. The phylogenetic tree was reconstructed with the neighbor-joining method with bootstrap replication in Molecular Evolutionary Genetics Analysis version 7 (MEGA7) [30], based on four protein datasets (DNA polymerase I, terminase large subunit, tail fiber protein and endolysin) to infer the evolutionary relationship of the studied bacteriophages and other bacteriophages from different families and genera. These four proteins have been utilized as phylogenetic markers in investigations of distantly related bacteriophages of *Enterobacteriaceae* [31,32].

## 3. Results

In this study, the eight bacteriophages were selected out of fifty-eight isolates [16] for characterization due to their ability to target the KP2 *Klebsiella* strain, a multi-antibiotic resistant (including carbapenems) with positive extended spectrum beta-lactamase (ESBL) and carbapenem-resistant-enzyme (CRE) profiles (not yet published). In addition, the two isolates exhibit broader infective spectrums in comparison to the other isolates.

### 3.1. Phage with Active Depolymerase Activity

**Figure 1:**
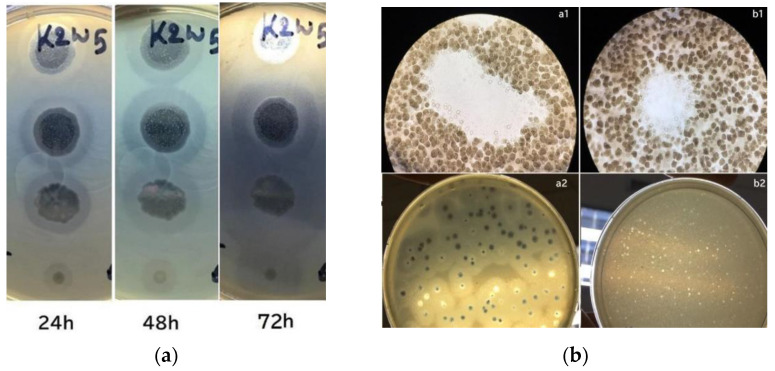
(**a**) Development of plaque halos around k2w5 plaques. The halos continue to expand after the central lysis area ceased to grow; (**b**) Bacteriophage plaques. (**a1**,**b1**) Optical microscope image of bacteriophage plaques and bacterial debris, on bacterial lawn. (**a2**,**b2**) A lawn of *K. pneumoniae* containing plaques after 4 and 16 h.

In this study, the phages and host were incubated for a prolonged period during plaque assay to observe their depolymerase activity. Large halos were observed on bacterial lawns and gradually increased their size over time. Figure 1a shows a lawn of *K. pneumoniae* containing plaques from K2w5 as an example. The size of k2w5 plaque increased by 0.5–1 mm and the halo size increased by 3–6 mm when plaques were further incubated for 72 h. As shown in Figure 1b(a1,b1), clearings (plaques) were formed on the bacterial lawns due to the inhibitory effect of the bacteriophages against the hosts. We did not observe bacterial growth within the plaques, indicating that the phages successfully killed the hosts. The bacteriophages exhibited varying levels of depolymerase activity as indicated by the variations in the diameters of the halos. We show the plaques with and without halos in Figure 1b(a2,b2), respectively.

The largest halo was observed to be formed by K9coc (Table 2). Whereas, k2a and k9w5 were presumed to have lower depolymerase activity due to the smaller halo diameter at 0.5 mm. The continuous increment in halo diameters over time indicates that these phages continue to produce highly active depolymerases over time [33]. This is due to the degradation of the extracellular polymeric structures, like exopolysaccharides, from the host strain [34].

**Figure 2:**
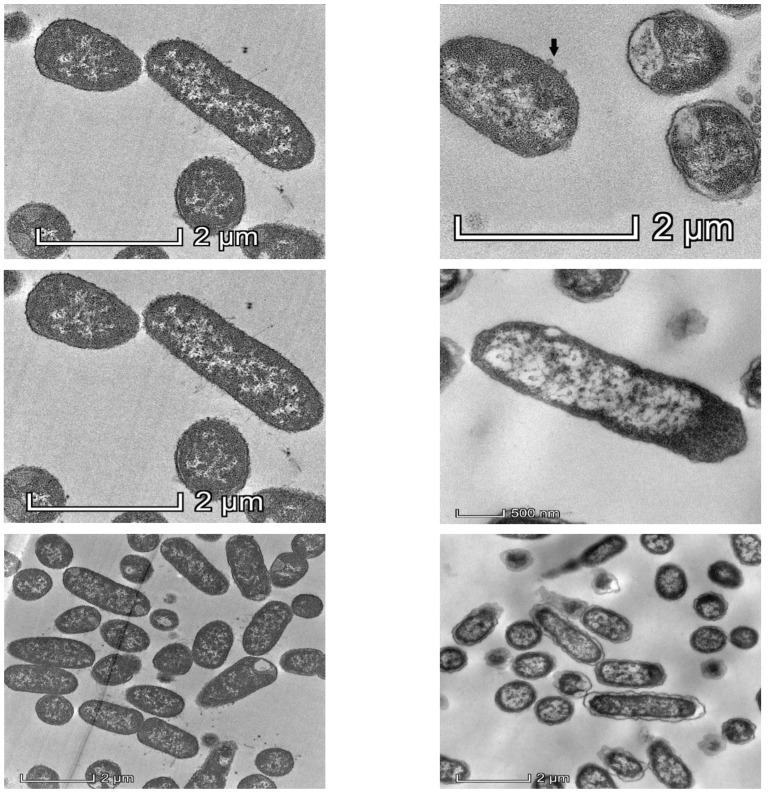
Representative TEM images of 50 nm sections of untreated control (**left panel**) and k9w6-treated *K. pneumoniae* (KP2) (**right panel**), (black arrow) show k9w6 attached to the bacteria wall. TEM images show weakened and disrupted cell wall after exposure to k9w6 at MOI 1 for 1 h.

Observation of *Klebsiella pneumoniae* under TEM showed drastic changes in intracellular density, disintegration of the cell wall and the cell membrane after 1 h exposure of KP2 to bacteriophage k9w6, as shown in Figure 2. The bacterial cells were observed to be enlarged. These changes suggest that k9w6 can effectively adsorb and penetrate the outer membrane. This observation is in agreement with [35], which reported that phages encode endolysins causing peptidoglycan degradation.

### 3.2. Low MOI Is Optimal for Bacteriophage Propagation

**Figure 3:**
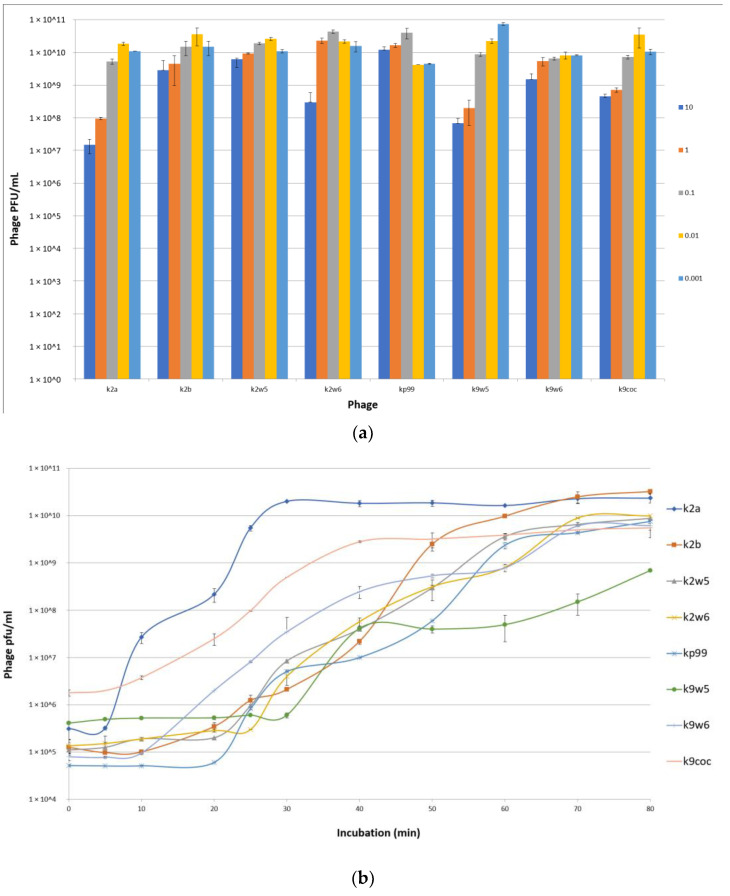

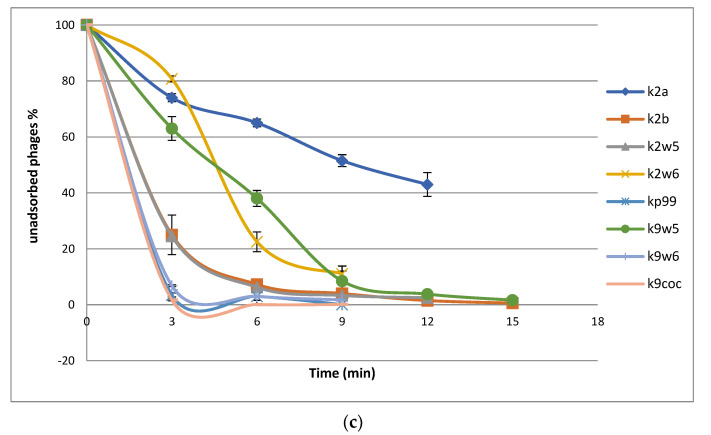
(**a**) Optimal multiplicity of infection (MOI) of bacteriophages. Comparisons of bacteriophage titers after incubation for 6 h at six different MOI (0.001, 0.01, 0.1,1 and 10 PFU/CFU); (**b**) The one-step growth curves of bacteriophages. The bacterial culture was mixed with the bacteriophage suspension to obtain MOI 0.01. Bacteriophages were allowed to adsorb for 10 min at 37 °C. Triplicate samples were taken every 10 min for 80 min and titrated. All data were collected from three independent experiments, error bars indicate the standard deviation; (**c**) The adsorption rate of the bacteriophages. Bacteriophages were mixed with excess *K. pneumoniae* KP2 cells, and the non-adsorbed infectious bacteriophages were serially counted. The data show percentages of unadsorbed bacteriophages relative to the initial input dose of bacteriophages.

An MOI of 0.01 was observed to produce the maximum bacteriophage titer for k2a, k2b, k2w5 and k9coc, signifying that 0.01 was the optimal MOI for the bacteriophage titer production. This MOI produced lysates of more than 2 × 10^10^ pfu/mL. The optimal MOI for k2w6 and kp99 bacteriophages were 0.1, 0.001 for k9w5, while k9w6 show similar concentrations in all tested MOI (Figure 3a). This finding is in accordance with [36,37], where low MOI was found to be optimal for the propagation and high bacteriophage stock preparation. Lysates products were optimum when a higher bacteriophage concentration was obtained [38].

### 3.3. One-Step Growth Curve

The efficiency of bacteriophage infection was characterized by observing its latent period (replication inside cell host) and burst size, which are the important characteristics of infection. Table 3 shows the burst size and latent period for the different bacteriophages under study. From Figure 3b, we see that the latent periods of the bacteriophages varied between 5 to 30 min, and the burst sizes of these bacteriophages varied between 30 to 354 particles per cell (PFU/cell). The k2a, k9coc and k9w6 bacteriophages had the shortest latent period (5–10 min) with more than 100 pfu/cell followed by bacteriophages K2b, k2w6 and k2w5.

The bacteriophages were reported to have a latent period with a range from 10–25 min and a burst size of 60–346 (PFU/cell) [39]. All phages were observed to have latent periods and burst sizes within the reported range, except for k9w5 (latent period > 25), K2a (latent period < 10) and K2b (burst size < 60). The larger latent period corresponded to the difficulty of obtaining a high stock concentration for the K9w5 bacteriophage. A higher stock concentration was obtained only when combining the liquid and agar propagation at the same time to get 10^10^ PFU/mL. The burst sizes of the isolated bacteriophages in this current study are considered large when compared to other *K. pneumoniae* bacteriophages, in which, the average burst size was as small as 50 pfu/cell [40]. Conversely, [41] reported a burst size of 9000 pfu/cell for a Podovirus bacteriophage phiAxp-3.

Differences in burst sizes and the latent periods of different bacteriophages depend on the nature of the bacteriophage, the physiological state of the host, the composition of the growth medium, the pH and the temperature of incubation. The smaller burst size may be caused by the large size of the bacteriophages and the small size of a host cell. The size of the host cell is crucial, as it modulates receptor availability and its protein-synthesizing machinery for the binding and growth of bacteriophages, respectively [42]. Larger burst sizes and longer latent periods increase the probability of a successful dispersal in the environment [43], which indicates that the bacteriophage is widely available for isolation.

### 3.4. Adsorption Parameters

The bacteriophage rate of adsorption was determined by titrating the unadsorbed phages against the initial titer. The number of free bacteriophages in the suspension declined over time, as shown in the adsorption curve (Figure 3c). Within the first 3 min, k2a, k2w6 and k9w5 have adsorption rates of less than 40%; k2w5 and k2b have adsorption rates of more than 70%; and the most efficient are k9coc (98%), kp99 (96%) and k9w6 (93.4%) (Figure 3c). A study reported a higher absorbance of *K. pneumoniae* (99.7%), but this is after 5 min of incubation [40]. Most bacteriophages were completely adsorbed after 12 min, except k2a. The high adsorption rate of bacteriophages in the current study is higher than what was reported in a recent study targeting the *Klebsiella* bacteriophage, which also belongs to *Podoviridae* family [44]. Different families of bacteriophages have different adsorption rates, as shown in several studies [45], because of either the absence or presence of extra side tail fibers or an improved tail fiber, contributing to rapid adaptation of the bacteriophage population [46].

### 3.5. Effect of Temperature on Bacteriophages

**Figure 4:**
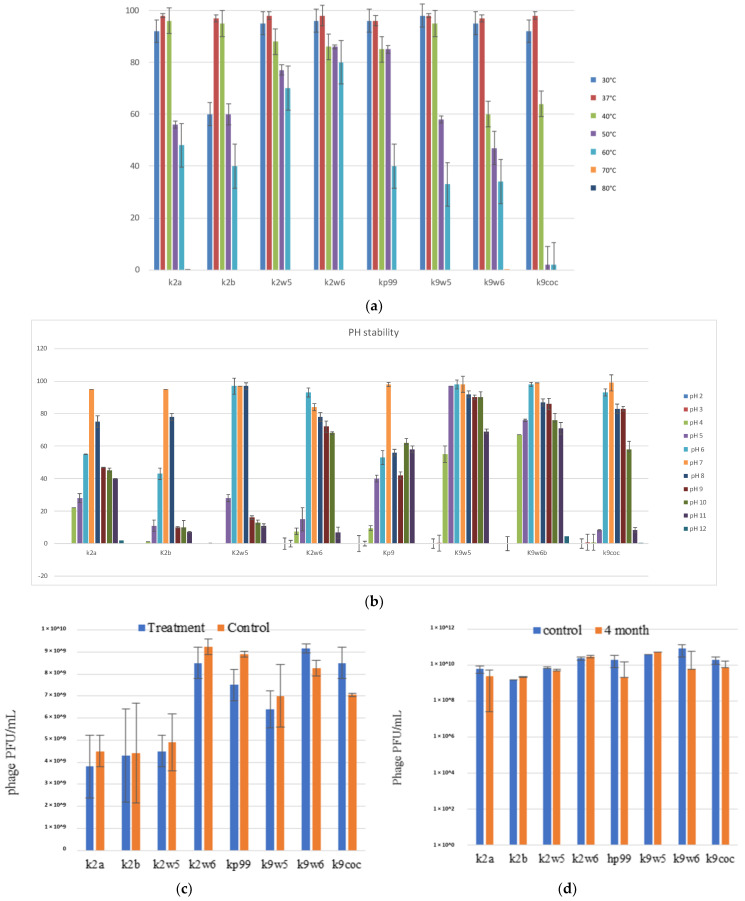
The stability of bacteriophages at different temperature, pH, chloroform, and long-term storage. (**a**) The fffect of variable temperatures on bacteriophage activities; (**b**) The effect of different pH on bacteriophage activities; (**c**) The effect of chloroform on the bacteriophage; (**d**) The effect of long-term cold storage on bacteriophage activities. All values represent the mean of three replicates ±SD.

The temperature is vital for bacteriophage growth in bacterial hosts and is an essential element to consider for maintaining bacteriophages over time [19]. All bacteriophages were observed to be viable between 30–60 °C, with a decrease of viability at higher temperatures (Figure 4a); k2w6 had the highest viability, with more than 80%, even at 60 °C, showing that it is the most thermo-stable, followed by k2w5; with more than 70% viability at the same temperature. The optimum temperature for all bacteriophages is 37 and 40 °C, except for k9w6 and k9coc. Another study reported the same finding, where the maximum infectivity was seen at 37 °C and 40 °C [47]. Since the bacteriophage depends on the host to propagate, the optimal temperature for the phage viability may correlate to the optimal temperature of its host. The most temperature-sensitive bacteriophage is k2b, where viability reduced drastically at lower (<37 °C) and higher temperature (>40 °C). All bacteriophages were unable to withstand temperatures ≥70 °C due to the high temperature-related denaturation of bacteriophage proteins [48]. Previously reported *Klebsiella* bacteriophages showed a 100-fold decline in titer when exposed to 60 °C at 10 min [40] and other studies reported complete inhibition at 60 °C [32,49,50,51]. Thus, bacteriophages k2w5 and k2w6 are novel in their ability to tolerate high temperature.

### 3.6. Effect of Different pH Values on Bacteriophages

Following the exposure to pH in range (2–12) for 1 h, all the bacteriophages demonstrated high tolerance to a wide range of pH (4–11), just as reported in another study [52]. They also showed maximum stability at pH 6–8, except for k2a, k2b and kp9, where viability was reduced to less than 60% at pH 6 for both k2a and k2b, while kp9 had reduced viability at all other pH, except for 7, indicating that the favorable pH for these bacteriophages was neutral, except for k2w6 (Figure 4b). *Klebsiella* phages with this range of stability have been reported before [47,53]. The most stable was k9w5, which showed viability of >90% at pH 5–10, with lowest viability (>55%) at pH 4. However, k9w6 had higher viability at pH 4 and 11 compared to k9w5. Nonetheless, both k9w5 and k9w6 exhibited higher pH stability as compared to previously reported bacteriophages [51,54,55].

All the bacteriophages were observed to still be viable at pH 4, with a reduction between 2 to 4-fold range, which indicates the potential to use these bacteriophages in the treatment of a *Klebsiella* infection when exposed to low pH conditions in the human gastrointestinal tract or acidic foods.

### 3.7. Effect of Chloroform on Bacteriophages

A neglectable reduction of bacteriophage activity was observed for k2a, k2w5, k2w6, kp99 and k9w5; on the other hand, there was a slight increment in k9w6 and k9coc bacteriophage concentration after treatment with chloroform. As a whole, chloroform exposure did not have any significant adverse effect on bacteriophages titers (Figure 4c). They remained unaffected by the 10% chloroform treatment after incubation for 1 h, just like bacteriophages in another study [40]. Based on these results, chloroform also did not render the bacteriophages inactive as they retained their plaque-forming ability. Small increases were obtained in k9w6 and k9coc concentration, which was regarded as an anomaly. A similar observation in a previous study was also reported [56]. These increases are not high and could derive from the dissociation of bacteriophage aggregates as discussed. The performance of bacteriophages sensitivity to chloroform was essential since bacteriophages are often treated with chloroform to preclude microbial contamination during storage [57].

### 3.8. Long-Term Storage of Bacteriophages

Bacteriophage lysate stocks were stored at 4 °C in sterile glass bottles that were covered in aluminum foil to preclude any light damage to the bacteriophage for four months to determine any reduction of bacteriophage concentration over the period. We found minimal reduction of bacteriophages concentrations ranging between 0.18–1.2 log (Figure 4d). A slight drop in the bacteriophage concentration could be attributed to the bacteriophage aggregation, causing the production of one plaque for several aggregated bacteriophages [58]. Storage at 4 °C was found to be the ideal temperature for maintaining bacteriophages over time. This result aligned with the findings from other studies, in which, 4 °C was described as the most favorable temperature for maintaining bacteriophage stocks [59,60]. Increases of bacteriophage concentrations were also noted for k2b and kp99, a phenomenon previously witnessed for *Bacillus thuringiensis* bacteriophages [61]. Another study showed elevated concentrations of up to 3 log changes for *Escherichia coli* and *Mycobacterium smegmatis* bacteriophages that had been stored for up to 2 years at 4 °C and 25 °C in liquid media that had been lyophilized [62].

### 3.9. Restriction Fragment Length Polymorphism (RFLP) of Bacteriophage DNA

**Figure 5:**
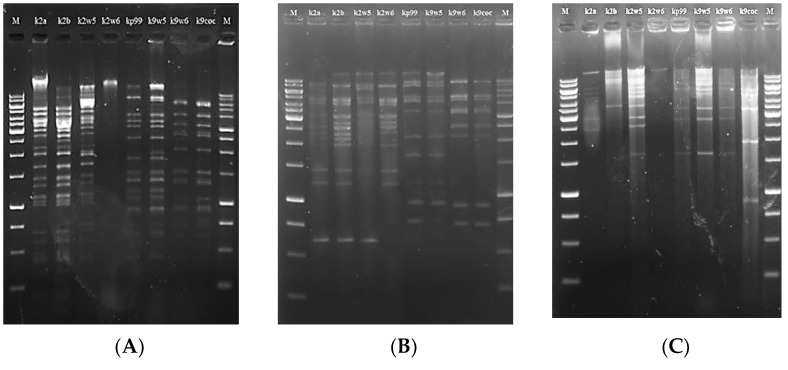
Restriction digestion analysis of the eight bacteriophages DNA. M:1 kb DNA marker, image (**A**) pstI, (**B**) smaI, (**C**) NdeI.

RFLP analysis has been used to assess genetic diversity among bacteriophages [58,63]. Six type II restriction enzymes: PstI, XhoI, XbaI, EcoRI, SmaI and NedI were used to restrict the DNA at specific sites (Table 4). These enzymes were chosen because several studies reported the susceptibility of bacteriophage genomes to them [64,65]. However, we observed that all eight bacteriophage DNAs were resistant to digestion with EcoRI, xbaI and XhoI, just as reported in previous studies [53,66]. On the other hand, PstI, NdeI and smaI enzymes successfully restricted the bacteriophage DNAs to yield sufficient discriminatory patterns (5 to over 20 bands), except for k2w6 (See Figure 5).

The evidence of digestion resistance may indicate that k2a, k2b, k2w5, kp99, k9w5, k9w6 and k9coc belong to similar genera, but not k2w6. Many bacteriophages possess anti-restriction mechanisms against host restriction endonucleases. These mechanisms include the restriction-inhibitory proteins, the modification of DNA via self-methylation or the activation of host methylase, and the lack of recognition sites for particular restriction enzymes [67,68].

The different banding patterns found after digestion with restriction enzymes corroborate the existence of dsDNA in all *Klebsiella* bacteriophages that are genetically similar. This is a defining characteristic of the tailed bacteriophages (*Caudovirales*). The sum of the fragments resulted in the genomic sizes of approximately 36–87 Kb (Table 3). The genome size of these bacteriophages is similar to the genome size of *Podoviridae* family that was reported in the range between 41.6 and 74.9 kb [69].

The three bacteriophages with comparatively largest genome sizes characterized in this study were 62, 64 and 87 kb, respectively. Kp99 and k9w5 shared the same genome size and restriction patterns despite belonging to different bacteriophage families. The same situation was reported in [70]. The remaining bacteriophages k2a, k9w6 and k9coc had smaller genome sizes, 36 kb, which was similar to the genome size of *Klebsiella* bacteriophages that was isolated by Can Wang et al. [71]. Interestingly, bacteriophages with higher similarity in their digestion profiles were also comparable in their lytic spectrum against the clinical *K. pneumoniae* KP2 strain. The genomic differences among these bacteriophages were clearly revealed using smaI, as it is possible to visualize either the presence or absence of some bands. For example, the band patterns in the region of 625 bp and 4000 bp were different among the bacteriophages. These different patterns paratition the eight bacteriophages into three groups (k2a, k2b, k2w5, k2w6), (kp99, k9w5) and (k9w6, k9coc). Notably, the first and the second group were more specific for the host of isolation, while the third group had a broader host range. This might be the product of the high degree of genome homology shared among bacteriophages with similar genome sizes [72]. This suggests that these bacteriophage particles are different but very closely related. Considering the similarities observed in these bacteriophages, they should undergo genomics analysis and sequencing to provide deeper insights into the relatedness observed in the present study.

### 3.10. Random Amplified Polymorphic DNA (RAPD)-PCR Analysis

**Figure 6:**
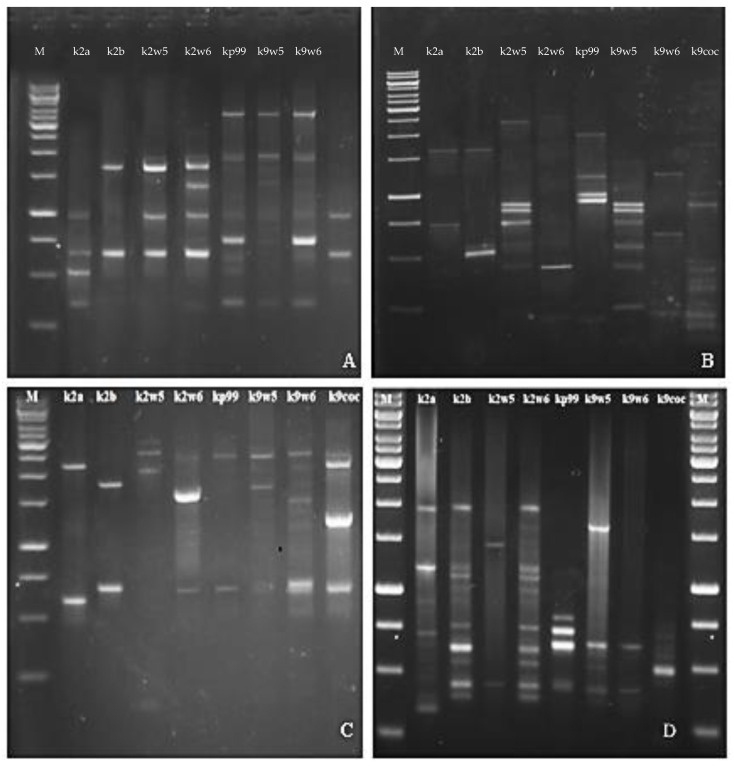
Agarose gel electrophoresis showing RAPD PCR amplification of DNA isolated from eight newly isolated *K. pneumoniae* phages with Primer (**A**) RAPD5, (**B**) R10, (**C**) RAPD2, (**D**) RAPD1.

The RFLP study was further validated with RAPD. RAPD-PCR is a method for quick typing of bacteriophage isolates and assessment of their genetic diversity [26]. Bacteriophages targeting a similar host but are diverse genetically would make a good cocktail [73] for therapeutic use as they reduce the probability of the host developing resistance to the phage. The primers used in this method generated band patterns with amplicons ranging in size from about 100 bp to 3.5 kb. The RAPD profiles of RAPD2 and RAPD5 gave high reproducibility values across bacteriophages tested for genomic fingerprints; on the other hand, R10 and RAPD1 produced distinct bands. A total of (3–7) bands were observed for primer R10 and (3–11) bands for RAPD1 (See Figure 6B,D). These two primers led to very different band patterns, which indicates the bacteriophages are genetically unique. In contrast, when primer RAPD5 and RAPD2 were used, similar bands were observed for bacteriophages that belonged to the same family. Identical RAPD patterns were found in two different series of experiments (Figure 6A,C).

**Figure 7:**
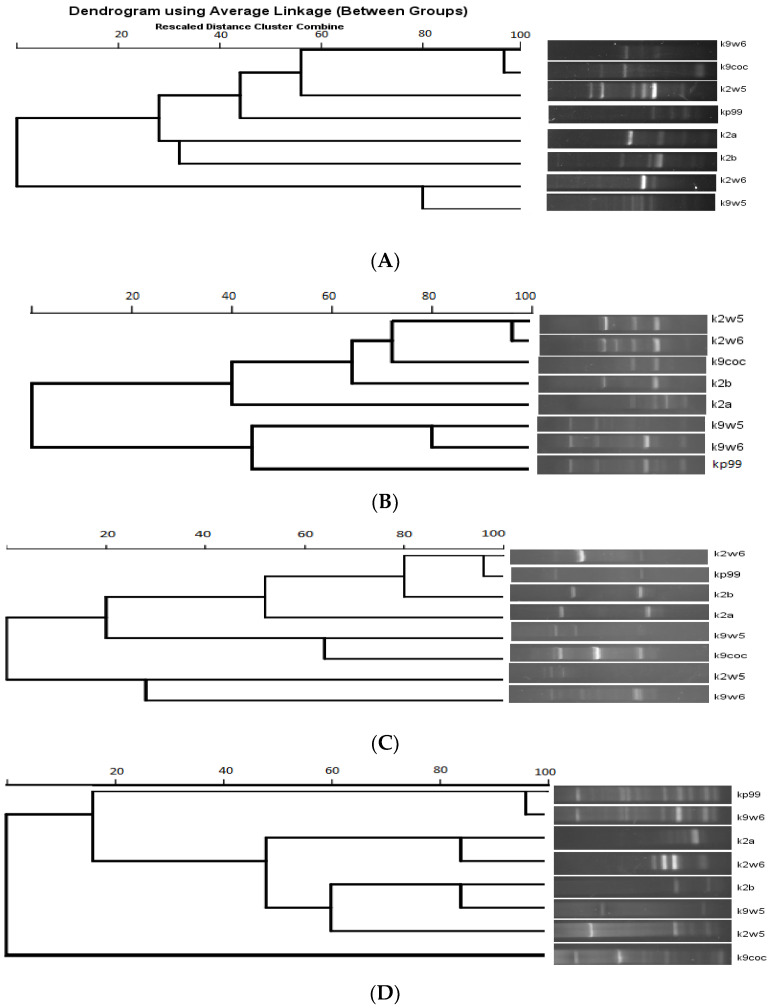
Dendrogram of eight *K. pneumoniae* bacteriophages. (**A**) RAPD primer R10. (**B**) RAPD primer 5. (**C**) RAPD primer 2. (**D**) RAPD primer 1.

Figure 7 shows the dendrogram analysis of the bands. Figure 7B displays two main clusters. These clusters largely group the bacteriophages that have the same host of isolation. For example, k9coc, k9w5 and kp99 clustered together and shared KP9 as a host of isolation, while k2a, k2b, k2w5 and k2w6 share KP2 as a host of isolation. No correlation was found between bacteriophage morphology and RAPD-PCR clustering, as all bacteriophages belonged to *Podoviridae,* except one (*Myoviridae*). Notably, the correlation between genome size and a low number of bands was observed; k2a had a small size and a low number of bands, while k2w5 (87 kb) generated a high number of bands with all the primers assayed (Figure 7A–D).

### 3.11. Analysis of Bacteriophage Structural Proteins

**Figure 8:**
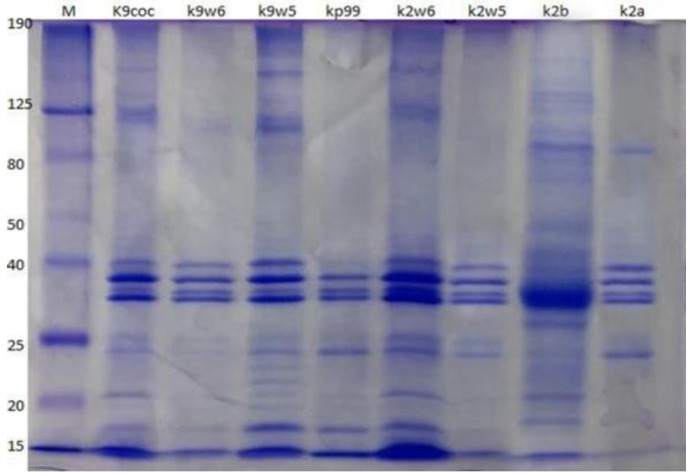
Bacteriophage analysis by SDS-PAGE. Samples were run on a 12% SDS-PAGE Tris-glycine gel, stained with Coomassie Brilliant Blue. The column labeled ‘M’ is a broad range protein molecular weight marker (HyperPage/Bioline). Estimated molecular masses are indicated on the left side.

Protein compositions of the eight bacteriophages were analyzed using SDS-PAGE. The bacteriophages have a similar protein profile, except the protein fractions with molecular weights of 22 and 24 kDa, which were observed only in k9w5 (*Myoviridae*). Four major protein bands were identified, out of at least ten clearly distinguished bands ranging from 15 to 190 kDa (Figure 8). These four most predominant polypeptide bands ranged between 32–40 kDa in size. These easily detected bands were most likely the major head and tail proteins [74]. A similar study found that the most predominant polypeptide band of about 35 kDa presumably corresponded to a major capsid protein [53]. Nevertheless, considerable variation is present in *Klebsiella* bacteriophages concerning protein size and number. For example, structural proteins of *K. pneumoniae* T7-like bacteriophages showed twelve bands with a broad range of sizes from 29 to 120 kDa [47]; while in another study, proteins of five isolated *Klebsiella* bacteriophages showed only two to five bands ranging from 20 to 55 kDa [75].

### 3.12. Phylogenetic Analysis

**Figure 9:**
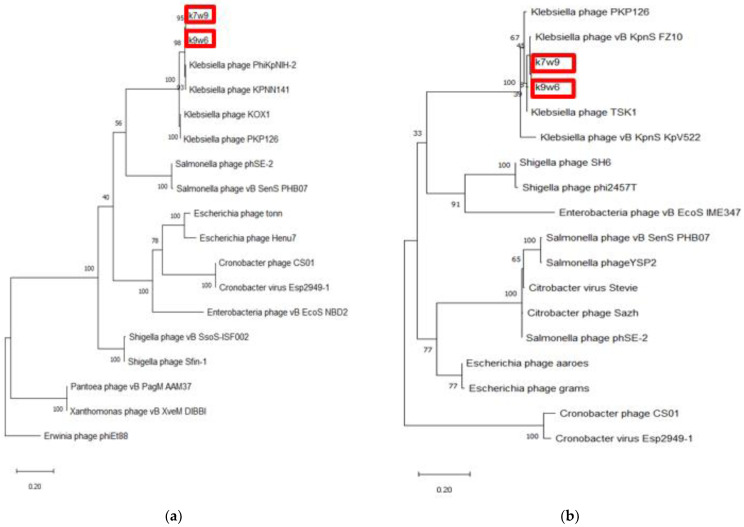
The phylogenetic tree of k9w6 and k7w9 the bacteriophage terminase large subunit and endolysin. The tree was constructed by using the maximum likelihood method with 2000 bootstrap replications. (**a**) The terminase large subunit of cronobacter bacteriophages was used as an out-group, (**b**) The Endolysin of Erwinia bacteriophage PhiEt88 was used as an out-group. The GenBank accession numbers are also provided after bacteriophage names, in parentheses. This tree showed the relationship of k9w6 and k7w9 terminase large subunit, endolysin with other close and distant bacteriophages.

Two bacteriophages were chosen for whole genome sequencing, k9w6 (monovalent) and k7w9 (uncharacterized, polyvalent bacteriophage), due to their broad host range, clear plaques and the size-enlarging haloes (depolymerase activity), to further investigate the difference between them at the genetic level. The DNA of k9w6 and k7w9 was extracted, genome sequencing was performed, and phylogenetic analysis was conducted with confident bootstrap values using four shared proteins (tail fibre protein, DNA polymerase, terminase large subunit and endolysin). The phylogenetic tree of the terminase large subunit and the endolysin protein shows that the sequences from k7w9 and k9w6 are most closely related to each other. The pair also clustered together with other *Klebsiella* bacteriophages with very high bootstrap support. In the terminase large subunit tree, the next closest sequences are from *Salmonella* bacteriophages, while sequences from *Escherichia*, *Cronobacter* and *Enterobacteria* bacteriophages comprise its adjacent cluster. Although in the endolysin tree, both bacteriophages cluster near to *Shigella* and *Enterobacteria* bacteriophages and afield from *Salmonella* (See Figure 9). A particular interest is focused on bacteriophage enzymes involved in the first step of viral infection responsible for bacterial envelope degradation, termed depolymerases, and on proteins encoded by lysis cassette genes, such as endolysins (peptidoglycan degrading activity) [40]. This was used successfully against gram-negative representatives, as well [76].

More than 25% of known dsDNA bacteriophages carry the DNA polymerase I (polA) gene, making the DNA polymerase I (polA) gene among the most broadly distributed bacteriophage genes. Due to its pivotal role in DNA replication, this enzyme is linked to characteristics of the bacteriophage lifecycle. It is a helpful marker for marking the diversity and composition of the virioplankton and could be a driving element in the divergence of bacteriophage replication components [77]. Again, by DNA polymerase tree, the k7w9 and k9w6 sequences were found to be most closely related to each other and also cluster closely with sequences from other *Klebsiella* bacteriophages (Figure 9). The next closest sequences in an adjacent cluster come from *Enterobacter* and *Shigella* bacteriophages. The hosts of these phages, alongside *Klebsiella*, are from the *Enterobacteriaceae* family of the *Enterobacterales* order. The next closely related sequence is from a bacteriophage of *Pantoea*, which is of the *Erwiniaceae* family of the same order.

**Figure 10:**
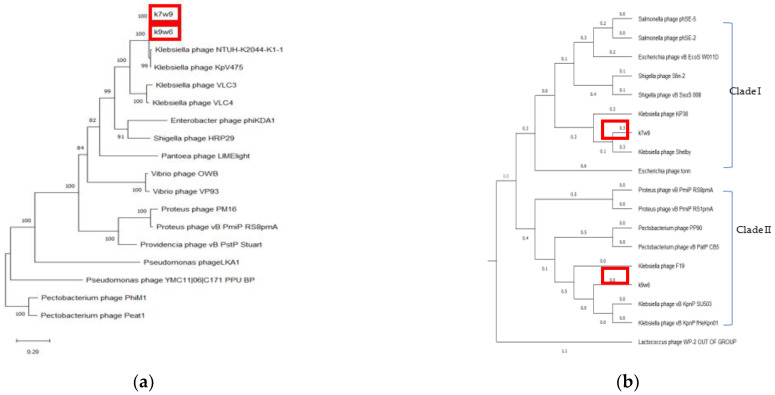
The phylogenetic tree of k9w6 and k7w9 bacteriophage (**a**) DNA polymerase, (**b**) Tail fiber protein. The tree was constructed by using the neighbour joining method with 2000 bootstrap replications. Pectobacterium and *Lactococcus* bacteriophage was used as an out-group. The GenBank accession numbers are also provided after bacteriophage names, in parentheses. This tree shows the relationship of k9w6 and k7w9 DNA polymerase and Tail fiber protein with other close and distant bacteriophages.

Bacteriophage tail fibers are elongated protein assemblies that can be recognized explicitly on bacterial surfaces during the first step of viral infection [78]. K9w6 and k7w9 bacteriophages were found to have different host ranges according to Clade I Clade II 185 monovalent and polyvalent action and different families, where k7w9 was related to the *Siphoviridae* family (data are not shown). To clarify these similarities, the tail fiber protein of k9w6 and k7w9 were compared with closely related *Klebsiella* and other bacterial bacteriophages proteins in the same phylogenetic trees. The analysis revealed that these two were separated into different monophyletic groups. Figure 10b shows the two clades. Clade I; comprises bacteriophages related to the *Podoviridae* family, k9w6 and *Klebsiella* bacteriophages involved in KP34-like viruses are clustered together in the tree, confirming that k9w6 is a podobacteriophage belonging to the Kp34-like virus genus *Autographiviridae* subfamily and is less similar to *Proteus* and *Pectobacetriu*. Clade II, including bacteriophages related to the *Siphoviridae* family, k7w9 is clustered next to (shelby) KP36 bacteriophage (siphoviruse) in the subfamily *Drexlerviridae* and is less similar to *E. coli*, *Salmonella* and *Shigella* bacteriophages. This suggests that bacteriophages in clade I or II may be closely related in terms of their evolution and have *Podoviridae* or *Siphoviridae* morphology. Broad- and narrow-host-range bacteriophages were found in all three families, *Myoviridae*, *Siphoviridae* and *Podoviridae* [70]. A similar result has been found where a variation in the tail fibre protein sequence associated to the host range differences between the two bacteriophages [79]. However, the detailed mechanisms underlying the interactions between binding proteins of the bacteriophage and receptors on the bacterial cell surface remains unknown. Although the host phylogenetic relation is not a definite indication of the bacteriophages being closely related, it is a point of reference as the possible evolution shift of a parasite usually results in an infection of species closely related to the natural host which supported host range result where k7w9 is polyvalent bacteriophage and infecting another bacterial species beside *K. pneumoniae* [16]. Most often, tail fiber and tail spike proteins display polysaccharide-depolymerase activity [80,81]. Some also have found that in several bacteriophages, the protein having the depolymerase domain was a part of bacteriophage structural proteins (mostly on tail fibers) and permits bacteriophage adsorption, invasion, and disintegration of the host bacterial cell [82]. Bacteriophages k9w6 and k7w9 were isolated from different sewage samples. The bacteriophages, as shown, have a different capsular specificity: k9w6 lyse *K. pneumoniae* of capsular type K2, and K57 and two strains of capsular K20, whereas bacteriophage k7w9 are lytic for strains of same capsular type, besides K53 and *S.aureus*, MRSA, *P.aeruginosa* and *B.subtillis*. The isolation of non-homology bacteriophages coding different tail fiber protein (degrading enzymes) from the different ecological environment shows that different environments contain different bacteriophages with possible isolation of new efficient bacteriophages with a broad host range. The DNA polymerase gene, terminase large subunit gene and Endolysin gene all supported that k9w6 and k7w9 were more closely related to each other than to other bacteriophages (no discrimination), but the observation of the tail fiber protein tree suggests that the tail fiber protein is a probable marker to identify the bacteriophages host range.

## 4. Limitation of This Current Study

Regarding the phage–host interaction, this study ended at in vitro testing of the phages’ activities against the bacteria. The study would be more complete with a systemic testing, as there could be unpredicted factors that would render the phages ineffective during treatment. For instance, the delivery method of these therapeutic microbes to infection sites and the ability of the phages to remain viable in vivo is unknown, and novel forms of resistance may materialize in the future. A large part of the variation in phage therapy is the in vivo pharmacokinetics and pharmacodynamics, making the application in the whole body system a challenge. This has also added to the lack of standardization, whereby different phages have their own variables in terms of burst, cycle and infectivity. Thus, although characterization of phage is important, in the future, studies on pharmacokinetics and pharmacodynamics will be critical to ensure the precise delivery of the therapeutic phages.

## 5. Conclusions

Carbapenem-resistant *Klebsiella pneumoniae* are highly pathogenic and are on the WHO list of AMR bacteria that are in urgent need for new antimicrobial or alternative therapies. Reported resistance towards last resort antibiotics such as colistin has made it inevitable to search for alternative treatments. Phage therapy is one of the alternatives that has recently been suggested by WHO. The bacteriophages used in this study have previously been described to be able to inhibit carbapenem-resistant *K. pneumoniae*. Their ability to produce depolymerases, which was observed in their plaques’ halo formations, also further increased their potential as therapeutic agents. The high stability shown by some of these isolates promises further potential applications as treatments. Nonetheless, further safety tests and detailed characterization, namely, regarding their effects against our immune system, need to be done before the phages can be utilized for therapeutic purposes in a clinical setup.

## Figures and Tables

**Table 1:** Primers used in RAPD-PCR; S and W represent G/C and A/T, respectively.

Primer	Sequence	Reference
R10D	5-GTCASSWSSW-3	[25]
RAPD5	5-AACGCGCAAC-3	[26]
RAPD2	5-CCGCAGCCAA-3	[26]
RAPD3	5-AACGGGCAGA-3	[26]
RAPD 1	5-GTAGACCCGT-3	[27]

**Table 2:** General characteristics of the eight bacteriophages.

Phage	K2a	K2b	K2w5	K2w6	Kp99	K9w5	K9w6	K9coc
Clear zone (mm)	1.5	1.5	4	6	1	0.5	1	1
Halo after 72 h (mm)	0.5	6.5	4	5	5	0.5	6	7

**Table 3:** The latent periods and burst sizes for different bacteriophages.

*K. pneumoniae* Bacteriophage	*K. pneumoniae* Host	Latent Period	Burst Size	MOI	Genome Size (kb)
K2a	KP2	5	116	0.01	36
K2b	KP2	20	41	0.01	62
K2w5	KP2	20	354	0.01	87
K2w6	KP2	25	106	0.1	62
Kp99	KP9	20	214	0.1	64
K9w5	KP9	30	66	0.001	64
K9w6	KP9	10	130	0.1	36
K9coc	KP9	10	210	0.01	36

**Table 4:** Endonucleases used for the restriction analysis of phage DNA.

Endonuclease	Restriction Site (5′ to 3′)
PstI	CTGCA/G
XhoI	C/TCGAG
XbaI	T/CTAGA
EcoRI	G/AATTC
SmaI	CCC/GGG
NedI	CA/TATG

## Data Availability

Not applicable.
